# Effect of Combined Balance Exercises and Kinesio Taping on Balance, Postural Stability, and Severity of Ankle Instability in Female Athletes with Functional Ankle Instability

**DOI:** 10.3390/life12020178

**Published:** 2022-01-26

**Authors:** Sara Mahmoudzadeh Khalili, Amir Hossein Barati, Rafael Oliveira, Hadi Nobari

**Affiliations:** 1 Department of Health and Sport Rehabilitation, Faculty of Sport Sciences & Health, Shahid Beheshti University, Tehran 19839-69411, Iran; s.mahmoodzadeh@mail.sbu.ac.ir or; 2 Sports Science School of Rio Maior-Polytechnic Institute of Santarém, Av. Dr. Mário Soares, 2040-413 Rio Maior, Portugal; rafaeloliveira@esdrm.ipsantarem.pt; 3 Research Center in Sport Sciences, Health Sciences and Human Development, Quinta de Prados, Edifício Ciências de Desporto, 5001-801 Vila Real, Portugal; 4 Life Quality Research Centre, Complexo Andaluz, Apartado 279, 2001-904 Santarém, Portugal; 5 Department of Physiology, School of Sport Sciences, University of Extremadura, 10003 Cáceres, Spain; 6 Sports Scientist, Sepahan Football Club, Isfahan 81887-78473, Iran

**Keywords:** FAI, injury, prevention, rehabilitation, sport, training, wobble board, women

## Abstract

Ankle sprain is a common musculoskeletal injury, and recurrent ankle sprains often lead to ankle instability. This study aimed to examine whether a 6-week balance training on a wobble board (WB) combined with kinesio taping (KT) is effective in improving balance, postural stability, and ankle stability among female athletes with functional ankle instability (FAI). Twenty-four female athletes with FAI were randomly assigned to study (SG) or control groups (CG). SG attended a 6-week training protocol of combined balance training on the wobble board with KT applied to ankles during exercise. CG only went through a 6-week balance training procedure that was the same as the SG. Before and after the training program, balance and postural stability and the severity of ankle instability were assessed by single-leg Biodex Balance system and Cumberland Ankle Instability Tool (CAIT), respectively. The analysis revealed that the scores of balance and postural stability decreased after the 6-week training for CG (*p* = 0.002) and SG (*p* = 0.001), which indicates an improvement for these variables, and the score of CAIT increased, which means the severity of instability reduced (*p* = 0.001 for both groups). Significant between-group differences were found for balance and postural stability (*t* = 2.79, *p* = 0.011, *g* = −1.99) and the severity of instability (*t* = 2.082, *p* = 0.049, *g* = 1.36), favoring SG compared with CG. This study showed that the addition of KT to balance training is more effective than balance training alone in improving balance, postural stability, and severity of ankle instability in female athletes with FAI. Our findings could provide a preliminary reference for designing combined balance and KT programs for delivering health benefits to females with FAI.

## 1. Introduction

Ankle injuries are common among athletes [1]. Ankle sprain is the most common musculoskeletal injury [2] and accounts for approximately 20% of sports injuries [3], with severe physical and economic consequences [4]. For example, after ankle sprain, 32–47% of patients report functional ankle instability (FAI) [5], a type of chronic ankle instability characterized by a tendency of the joint to sprain laterally and a feeling of “giving way” [6]. Female athletes have been shown to be more prone to ankle sprains than their male counterparts [7]. In functional instability, the joint moves far beyond the individual’s voluntary control in the physiological range [8,9]. In FAI, the anatomical structure of the joint does not change but results in proprioception and functional changes in the sensorimotor system of the ankle. Individuals with FAI are more prone to injury due to impaired postural control, balance, and proprioception [10,11].

The nervous system is responsible for controlling body position and movements through feedback and feed forward mechanisms. It is well established that dynamic stability of the ankle joint is usually controlled by the feed forward mechanism, which uses the visual, auditory, and tactile senses, as well as previous experience and internal models, to maintain body position and movements [3,12,13,14]. The changes in neuromuscular control could affect postural stability in patients with chronic ankle instability [15]. Postural stability is the ability to maintain the center of mass within the base of support, and it requires proprioception, neuromuscular control, and integration of somatosensory, visual, and vestibular afferent information [16].

It has been shown that with FAI, feed forward neuromuscular control is more important and can be influenced by exercise interventions [17,18]. Balance exercises are one of the significant interventions to reduce ankle injuries and rehabilitation [14,19]. Wobble or balance board exercises are usually effective in the rehabilitation of those with ankle instability problems [20]. These exercises can improve proprioception and the function of the joint mechanical receptors and improve performance and sense of stability in individuals with FAI [21]. In addition to exercise therapy, the use of various types of external supports such as braces and kinesio taping (KT) are also very effective [22,23]. KTs are cotton strips that are highly compatible with the skin and can stretch several times the original length. In addition to low cost, convenience, and accessibility, these tapes relieve muscle fatigue, increase blood circulation, reduce edema and swelling, increase range of motion, relieve pain, and increase joint awareness [24].

Based on the existing background literature, most studies have been conducted on male athletes in organized sports and considering that females and children are at the highest risk for suffering an ankle sprain [2], there is a need for more studies on this population. Moreover, since the results of studies [25,26] on the effects of KT are highly inconsistent, and considering that balance and postural stability are examined separately, this research examined: (1) the effect of 6-week balance exercises on WB in balance, postural stability, and severity of ankle instability of female athletes with FAI; (2) the effect of KT in balance, postural stability, and severity of ankle instability of female athletes with FAI; and (3) the difference between balance exercises alone and balance exercises plus KT. We hypothesized that the combined balance exercises and KT are effective in improving balance and postural stability and decreasing the severity of ankle instability.

## 2. Materials and Methods

### 2.1. Participants

Twenty-four female academy athletes of Shahid Beheshti University were randomly assigned into a study group (SG, balance exercises plus KT) and a control group (CG, balance exercises). A computer-generated random table was used for randomization. The study was between April and June 2018. All participants were required to meet the entry criteria. The ankle joint functional assessment tool (AJFAT) was used, which is known to be a reliable tool for detecting FAI [27]. Each item of the questionnaire was assigned a score ranging from 0 to 4, and the total score was 48. The higher the score, the higher the functional instability [28]. The inclusion criteria for participants were as follows: (1) collegiate athletes from the sports of volleyball, handball, or basketball who were regularly engaged in their daily activities; (2) history of at least one significant ankle sprain that required rest and immobility for some time; (3) feeling of ankle instability and “giving way” during daily routine and sports activities; (4) AJFAT score ≥26 [29,30]; and (5) fully able to tolerate body weight and walk normally with a complete range of motion of the ankle. Participants who had pain and swelling, underwent any kind of intervention (e.g., medicine, braces, and special shoes), received physical therapy before participating in the study, and were not present for two consecutive training sessions were excluded [31,32,33]. After signing the voluntary informed consent form, all participants were informed about the purpose of the study and the procedure. The study was approved by the research committee of Shahid Beheshti University (protocol code IR. 9010806-03) and followed the ethical guidelines of the Helsinki Declaration for Human Research.

### 2.2. Sample Size

G-Power software (University of Düsseldorf) was used to calculate sample power [34]. A priori sample power analysis was examined based on the *t*-test, difference between two dependent means (matched pairs), α error prob level = 0.05, effect size = 0.6 [35], and 1-β error prob level = 0.85. With 24 participants, the current analysis has an actual power of 85.9% [36].

### 2.3. The Pre-Test Assessment

The pre-test included measurement of anthropometric characteristics (i.e., height (Seca height gauge 207, made in Germany), weight (Seca electronic weighing 767, made in Germany), and body mass index (weight/height^2^)), assessment of the severity of ankle instability by the Cumberland Ankle Instability Tool (CAIT), and assessment of the balance and postural stability by the Biodex Balance SD System single-leg test (made in the USA).

### 2.4. The CAIT

The CAIT is a self-reported outcome questionnaire and has been adapted cross-culturally and translated to several languages, including Persian [37]. It has been shown to have the validity and reliability to assess the severity of ankle instability [38]. It can detect FAI and determine the severity of the instability [39] and includes nine multiple-choice questions. The answers to these questions are summed to give a total score between 30 (stable ankle) and 0 (extreme functional ankle instability). The lower the CAIT score, the more severe the ankle instability. Participants completed the questionnaire before and after the study.

### 2.5. Biodex Balance System, Single-Leg Test

The athletes’ balance and postural stability were checked with the Biodex Balance SD System both before and after the study [40] after a rest day and before training on that day. This device can quantitatively measure the balance and postural stability of participants and provide objective and comparable results. The Biodex Balance SD System is designed to stimulate the mechanical receptors of the joints and upgrade the necessary reflex activity of the muscles to stabilize the joint [41]. This device has sufficient validity to assess balance [42], and it is also reported to be a reliable device to assess changes in postural control [43].

The assessment of both groups was performed on the unstable ankle without KT. Athletes were asked to stand single-legged with eyes open in the center of the locked platform of the Biodex Balance SD System. They were asked to place both hands on their waist and maintain a slight knee flexion (≈0°). Level 8 (stable) to 6 (moderate) was considered in terms of athlete safety and the fact that lower levels fluctuate more [44]. Three 25-s tests with a 30-s resting interval were performed, and the overall score was considered. The device provides three stability indices: overall stability index, anterior-posterior stability index, and medial-lateral stability index. It has been shown that the overall stability score is the best index to indicate the overall ability of people with ankle instability to maintain balance [45]. The intraclass coefficient was calculated based on the test–retest reliability results, and it was 0.81 [46].

### 2.6. WB Balance Exercises Protocol

Both groups performed selected WB exercises three times per week for 6 weeks. Based on a similar study [47] and the characteristics of the participants, a progressive training protocol was designed (See Table 1).

**Figure 1:**
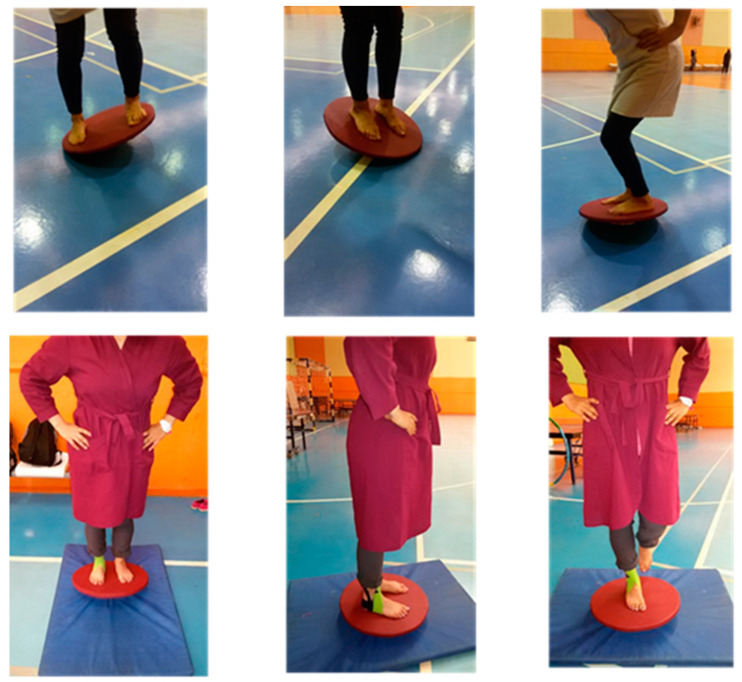
Balance exercises on wobble board.

During the exercises, the researcher used a chronometer to monitor time. The exercises were performed progressively, changing from open to the closed eyes and from a hard to a soft surface. During the 6 weeks, the CG performed the same balance exercises three times a week without the KT (Figure 1).

### 2.7. KT Technique1

**Figure 2:**
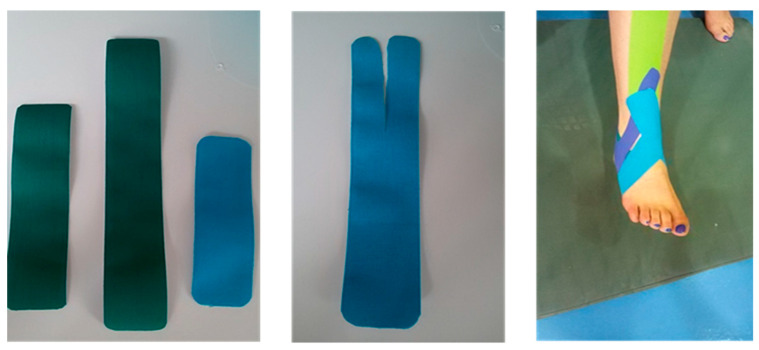
Kinesio taping technique.

The study group performed the exercises three times a week, while the researcher applied KT to their ankles. The researcher had a valid certificate for KT from the University of Tehran. The tape in each pack was 5 m long and 5 cm wide. Before taping, the taping area should be shaved and wiped with alcohol. Three tapes, two I-shaped and one Y-shaped, were applied within the first 3 weeks. The corners of the tapes were rounded to avoid any skin irritation. Due to the lateral ankle sprain, the direction of the tapes was from the inside to the outside, and the ankle was slightly in dorsiflexion. From the fourth week, another strip was added to the previous ones (Figure 2).

The starting point of one of the I-shaped KT was at the origin of the tibialis anterior muscle. Another I-shaped strip was placed from the medial malleolus to the lateral malleolus in the transverse direction, and the Y-shaped strip was placed from the medial malleolus to the sides of the lateral malleolus. The initial and final anchorages of KT were applied with a tension of 0%, while the therapeutic zone had a tension of approximately 30% in the first week. This tension was gradually increased to 80% during the last week. At the end of each session of the exercises, KT was removed. To activate the KT adhesive, the strip should be applied at least 20 min before the exercises [46]. Participants then performed exercises on the WB (with a diameter of 40 cm). At the end of the intervention period, the post-test was performed 24 h after the end of the 6-week exercise program to assess the effect of KT [24]. The post-test was completely similar to the pre-test.

### 2.8. Statistical Analysis

Means and SDs were used to report data. Normality of outcome values was tested using the Shapiro–Wilk test. The paired dependent and independent samples *t*-tests and covariance analysis were used to test the hypotheses. The magnitude effect size between the pre- and post-tests was estimated using Hedge’s *g* with a confidence interval of 95%. To assess the practical differences between groups, Hedge’s *g* effect size (ES) was used. The ES statistical thresholds were <0.2 for trivial, ≥0.2 to <0.6 for small, ≥0.6 to <1.2 for moderate, ≥1.2 to <2.0 for large, ≥2.0 to <4.0 for very large, and ≥4.0 for extremely large [48]. Results were analyzed with a significance level of *p* ≤ 0.05 using SPSS v25 for Windows.

## 3. Results

Both SG and CG showed no significant difference and were at the same level of the research variables before starting the exercises (Table 2).

The results of the paired *t*-test showed that in SG, there was a significant improvement in the post-test scores for balance and postural stability (*t* = 7.22, *p* = 0.001, *g*= −1.99, ES, large) and for the severity of instability (*t* = 6.73, *p* = 0.001, *g*= −1.36, ES, large). The balance and postural stability scores showed a significant difference in the SG compared with CG (*p* = 0.011), and there was also a significant difference in the severity of instability scores in SG compared with CG (*p* = 0.049) (Table 3).

## 4. Discussion

The main objective of this study was to investigate whether 6 weeks of balance exercises plus KT had any effect on balance, postural stability, and severity of ankle instability in female athletes with FAI. Our findings revealed that although balance exercises on WB had positive effects on improving balance and postural stability and reducing the severity of ankle instability in female athletes with FAI, accompanying KT with balance exercises had a greater effect on the aforementioned variables in this group of individuals.

Balance training is an efficient way to decrease recurrent ankle inversion in individuals with FAI [49], and both 3-week [49] and 6-week balance training protocols [50] could improve postural stability. Similarly, it has been shown that balance and plyometric exercises can enhance postural stability and reduce weakness caused by various constraints [51]. In addition, research findings indicated that WB exercises could improve poor dynamic and static balance caused by chronic ankle instability [52]. Another study investigating the effect of 4-week balance board exercises in subjects with FAI showed that this program improved the sense of stability [47]. However, one study indicated that the results of the research review were not strong enough to conclude with certainty that balance exercises lead to an improvement in postural control in people with FAI [53]. In this regard, the study by Ly et al. showed that postural control performance did not experience significant improvements immediately after KT or after 24 h [54]. Nevertheless, our study demonstrated significant changes in subjects’ balance and postural stability before and after 6 weeks of balance exercises on WB.

A group of researchers investigated the effect of ankle KT on postural stability in athletes with and without FAI and claimed that KT significantly improved the overall and side stability indices of both groups when standing on one leg. However, this effect was not observed in anterior-posterior stabilization indices in open and closed eye status [55]. Sarvestan et al. indicated that KT might help ankle stability by limiting the ankle joint range of motion and reducing the activity of gastrocnemius and peroneus longus muscles [56]. In a study that examined the effect of KT on the neuromuscular performance of femoral quadriceps, postural balance, and lower limb function in healthy subjects immediately after tape application, it was found that KT failed to positively affect the aforementioned factors [57]. In another study that focused on the immediate effects of KT on the neuromuscular performance of quadriceps and balance in subjects who had undergone anterior cruciate ligament reconstruction, they concluded that KT did not alter the neuromuscular performance of quadriceps and balance in this group [58]. Ingles et al. indicated that KT functional correction technique did not improve the dynamic and static balance of male amateur soccer players, whereas balance exercises combined with KT significantly improved these variables [59]. The present study showed that the balance and postural stability of both groups were statistically significant before and after the 6-week balance exercises plus KT.

Momeni-Iari et al. showed that immediately after KT, the absolute error of joint position sense, sense of force, and postural control decreased in subjects with FAI, and joint proprioception also improved [60], while Shield et al. found no improvement in specific postural control deficits in subjects with an unstable ankle after 24 h of continuous wearing of KT [25]. Similarly, Yin et al. dedicated the limited effect of KT to facilitate postural control in subjects with a chronic ankle sprain during the sensory organization test, unilateral stance, and limit of stability [26]. In addition, in a study that investigated the immediate effects of ankle balance taping with KT on the dynamic balance of young players with FAI, it was reported that ankle taping could improve dynamic balance [61]. Likewise, the results of a study on the effect of leg spiraling KT on postural sway in patients with multiple sclerosis showed that this technique could slightly improve postural sway in acute patients [62]. However, in a study that investigated the immediate and long-term effects of KT on balance and functional performance, no significant difference was found in balance and functional performance during calf muscle KT [63]. Even so, the present study also showed improvements in using balance exercises combined with KT.

### Limitations

One of the limitations of this study was the investigators’ blindness of group assignment and potential biases. Furthermore, the post-test was performed 24 h after the end of the 6-week training program [25], so the long-term effects of KT and the balance exercises could not be determined. In addition, only individuals with FAI were selected for this study. Therefore, it is suggested that future studies perform the same procedure in male subjects and individuals with mechanical ankle instability. Likewise, we could not compare the differences between healthy subjects and subjects with FAI, so the results are limited to the latter group only. Finally, since the physiological effects of KT are generally unobservable and the changes could also be related to psychological effects, we strongly recommend that these variables can be investigated in future studies.

## 5. Conclusions

The results of this study identified that balance exercises on WB combined with KT enhanced balance and postural stability and reduced the severity of ankle instability in female athletes who had FAI, and it has a more significant effect than solitary balance exercises. Although male athletes and other types of ankle instability were not considered in this study, it might be advisable that coaches, practitioners, and the individuals who become involved in female athletes’ rehabilitation include progressive balance exercises on WB plus KT to assist athletes with FAI to recover their balance and postural stability and decrease the severity of the ankle instability.

## Figures and Tables

**Table 1:** Balance exercises on wobble board protocol.

Eyes Status—Surface	Sets and Reps—Rest (between Sets)	Exercise	Session
**Eyes open—Hard Surface**	3 sets, 10 s-10 s rest	Maintain balance on wobble board	1
**Eyes open—Hard Surface**	1 × 30 s + 3 × 30 s-10 s rest	Maintain balance + forward and backward motion of the wobble board on both legs	2
		Repeat the exercises of the second session	3
**Eyes open—Hard Surface**	1 × 30 s + 3 × 30 s + 3 × 30 s + 1 × 30 s-10 s rest	Maintain balance + forward and backward motion + move the wobble board to the sides + circular movement of wobble board from front to side	4
		Repeat the exercises of the fourth session	5
**Eyes open—Hard Surface**	Each exercise (3 × 30 s)-10 s rest	Repeat the exercises of the fourth session in a semi-squat position	6
**Eyes open—Hard Surface-**	First 4 exercises (1 ×30 s) + last exercise (6 × 10 s)-10 s rest	Maintain balance + forward and backward motion + move the wobble board to the sides + circular movement of wobble board from front to side + standing with FAI leg on the wobble board and maintaining balance	7
		Repeat the exercises of the seventh session	8
**Eyes open—Hard Surface**	1 × 30 s+ Next 3 exercises (2 × 30 s) + last exercise (6 × 10 s)-10 s rest	Maintain balance + forward and backward motion + move the wobble board to the sides + circular movement of wobble board from front to side + standing with FAI leg on the wobble board and maintaining balance	9
**Closed eyes—Hard surface**	2 × 30 s-10 s rest	Maintain balance	10
**Closed eyes—Hard surface**	1 × 30 s + 3 × 30 s-10 s rest	Maintain balance + forward and backward motion of the wobble board on both legs	11
**Closed eyes—Hard surface**	1 × 30 s + next 2 exercises (3 × 30 s)-10 s rest	Maintain balance + forward and backward motion + move the wobble board to the sides	12
**Closed eyes—Hard surface**	1 × 30 s + next 2 exercises (3 × 30 s) + last exercise (1 × 30 s)-10 s rest	Maintain balance + forward and backward motion + move the wobble board to the sides + circular movement of wobble board from front to side	13
**Closed eyes—Hard surfaceIn semi-sitting position**	1 × 30 s + next 2 exercises (3 × 30 s) + last exercise (2 × 30 s)-10 s rest	Maintain balance + forward and backward motion + move the wobble board to the sides + circular movement of wobble board from front to side	14
**Closed eyes—Hard surface**	6 × 10 s-10 s rest	Standing with FAI leg on the wobble board and maintaining balance	15
**Eyes open—Soft Surface**	Each exercise (1 × 30 s)-10 s rest	Maintain balance + forward and backward motion + move the wobble board to the sides	16
**Closed eyes—Soft surface**	Each exercise (1 × 30 s)-10 s rest	Maintain balance + forward and backward motion + move the wobble board to the sides + circular movement of wobble board from front to side	17
**Closed eyes—Soft surface**	6 × 10 s-10 s rest	Standing with FAI leg on the wobble board and maintaining balance	18

Abbreviation: FAI, functional ankle instability.

**Table 2:** Descriptive information for groups.

Groups	BMI (Kg/m^2^)	Age (yrs)
**SG**	22.61 ± 2.91	24.8 ± 2.6
**CG**	21.88 ± 2.18	25.6 ± 2.5

Data are presented in mean ± standard deviation: SG, study group; CG, control group; BMI, body mass index.

**Table 3:** Between- and within-groups differences in balance and postural stability, and severity of instability before and after the intervention.

Assessments	Groups	Pre- Intervention	Post- Intervention	Within Groups		Between Groups				CI95% for Difference	Hedge’s *g* (95% CI)
				*t*	*p*	*t*	*p*	*t*	*p*	Values [Lower-Upper]	Values [Lower-Upper]
**Balance and** **postural stability**	BTG	1.38 ± 0.51	0.89 ± 0.21	3.95	0.002 ^a^	−2.07	0.051	2.79	0.011 ^b^	−0.49[−0.82 to −0.16]	−1.21[−2.08 to −0.34] L
	BT + KTG	1.88 ± 0.62	0.86 ± 0.22	7.22	0.001 ^a^					−1.01[−1.42 to −0.6]	−1.99[−2.96 to −1.01] L
**Severity of** **instability**	BTG	20.67 ± 2.39	23.42 ± 2.71	−5.4	0.001 ^a^	0.44	0.667	2.082	0.049 ^b^	2.74[0.58 to 4.9]	1.03[0.18 to 1.89] M
	BT + KTG	20.08 ± 3.97	24.58 ± 3.8	6.73	0.001 ^a^					5.5[2.21 to 8.79]	1.36[0.48 to 2.25] L

Data are presented in mean ± standard deviation: BTG, balance training group; BT+KTG, balance training + kinesio taping group; L, large; M, moderate; Hedge’s *g* (95% CI), Hedge’s *g* effect size magnitude with 95% confidence interval; ^a^ demonstrated significant results compared with the pre-test, at a significance level of ≤0.05; ^b^ demonstrated significant difference between groups before or after intervention at a significance level of ≤ 0.05.

## Data Availability

The datasets used and/or analyzed during the current study are available from the corresponding author on reasonable request.
